# A new genus and two new species of oonopid spiders from Tibet, China (Araneae, Oonopidae)

**DOI:** 10.3897/zookeys.1052.66402

**Published:** 2021-07-30

**Authors:** Weihua Cheng, Dongju Bian, Yanfeng Tong, Shuqiang Li

**Affiliations:** 1 Life Science College, Shenyang Normal University, Shenyang 110034, China Shenyang Normal University Shenyang China; 2 CAS Key Laboratory of Forest Ecology and Management, Institute of Applied Ecology, Shenyang 110016, China Institute of Applied Ecology, Chinese Academy of Sciences Shenyang China; 3 Institute of Zoology, Chinese Academy of Sciences, Beijing 100101, China Institute of Zoology, Chinese Academy of sciences Beijing China

**Keywords:** Asia, goblin spiders, morphology, taxonomy

## Abstract

A new genus, *Paramolotra* Tong & Li, **gen. nov.**, including two new species, *Paramolotra pome* Tong & Li, **sp. nov.** (♂♀) and *Paramolotra metok* Tong & Li, **sp. nov.** (♂♀), is described from Tibet, China. Morphological descriptions and photographic illustrations of the two new species are given.

## Introduction

Oonopidae Simon, 1890 is a diverse spider family with 1884 extant described species in 114 genera (WSC 2021). They are small spiders (usually < 3 mm), generally living in leaf litter (e.g., Dupérré et al. 2020), in canopies (e.g., Fannes et al. 2008; Tong and Li 2011), caves (e.g., Chamberlin and Ivie 1938; Tong and Li 2013). Some are termite nest inquilines (Benoit 1964) or even ant-mimics (e.g., Platnick and Dupérré 2011; Sun et al. 2019). Currently, 13 genera and about 100 species are known to occur in China (Li 2020).

The oonopid spiders of Tibet have been poorly studied. Hitherto, only two species, *Gamasomorpha linzhiensis* Hu, 2001 and *Ischnothyreus linzhiensis* Hu, 2001, have been recorded from Tibet (Hu 2001). In this paper, a new genus with two new species are proposed from material collected from Tibet are described and illustrated.

## Materials and methods

The specimens were examined using a Leica M205C stereomicroscope. Details were studied under an Olympus BX51 compound microscope. Photos were made with a Canon EOS 550D zoom digital camera (18 megapixels) mounted on an Olympus BX51 compound microscope. Vulvae were cleared in lactic acid. For scanning electron microscopy (SEM), specimens were air-dried, sputter coated using IXRF SYSTEMS, and imaged with a Hitachi TM3030 SEM. All measurements were taken using an Olympus BX51 compound microscope and are in millimeters. The type material is deposited in Shenyang Normal University (SYNU) in Shenyang, China (curator: Yanfeng Tong).

The following abbreviations are used in the text and figures: ALE = anterior lateral eyes; am = anterior membrane; ami = anterior median indentation; ap = anterior protrusion; apo = apodemes; bp = basal protrusion; csp = cone-shaped protuberance; dp = distal protrusion; lt = large tooth; PLE= posterior lateral eyes; pm = posterior membrane; PME = posterior lateral eyes; pp = posterior protrusion; pr = posterior receptaculum; ss = slit sensillum; tls = tube-like structure; tsc = T-shaped sclerite.

## Taxonomy

### Family Oonopidae Simon, 1890

#### Paramolotra

Taxon classificationAnimaliaAraneaeOonopidae

Tong & Li gen. nov.

E020B25E-8EC7-5E96-AE21-BA98E7789712

http://zoobank.org/B2E12C65-8654-427E-A058-7F78B39D8EE9

##### Type species.

*Paramolotra pome* sp. nov.

##### Etymology.

The generic name refers to the similarities of this genus with *Promolotra* Tong & Li and is feminine in gender.

##### Diagnosis.

*Paramolotra* gen. nov. resembles *Promolotra* Tong & Li, 2020 in having the heavily sclerotized dorsal and ventral abdominal scuta, the long spines on legs I and II, the cone-shaped protuberance on anterior face of male chelicerae, and the completely fused bulb and cymbium, but can be distinguished by the embolar region which have several protrusions that distinctly extend beyond the tip of the cymbiobulbus (Figs 2, 5, 7A–D), the indented labium (Fig. 8A, G) of both sexes, and the stick-shaped anterior arm of T-shaped sclerite (tsc) of endogyne (Fig. 9B, D). The embolar region of *Promolotra* consists of brush-like structures and 3 broad lobes, which barely extends beyond the tip of the cymbiobulbus (Tong and Li 2020: figs 1H–J, 2, 5H–J, 6), the labium deeply incised (Tong and Li 2020: fig. 3E, 7E), the endogyne consists of canopy-shaped anterior arm of T-shaped sclerite (tsc) and horseshoe-shaped sclerite (Tong and Li 2020: fig. 4G).

**Figure 1.**
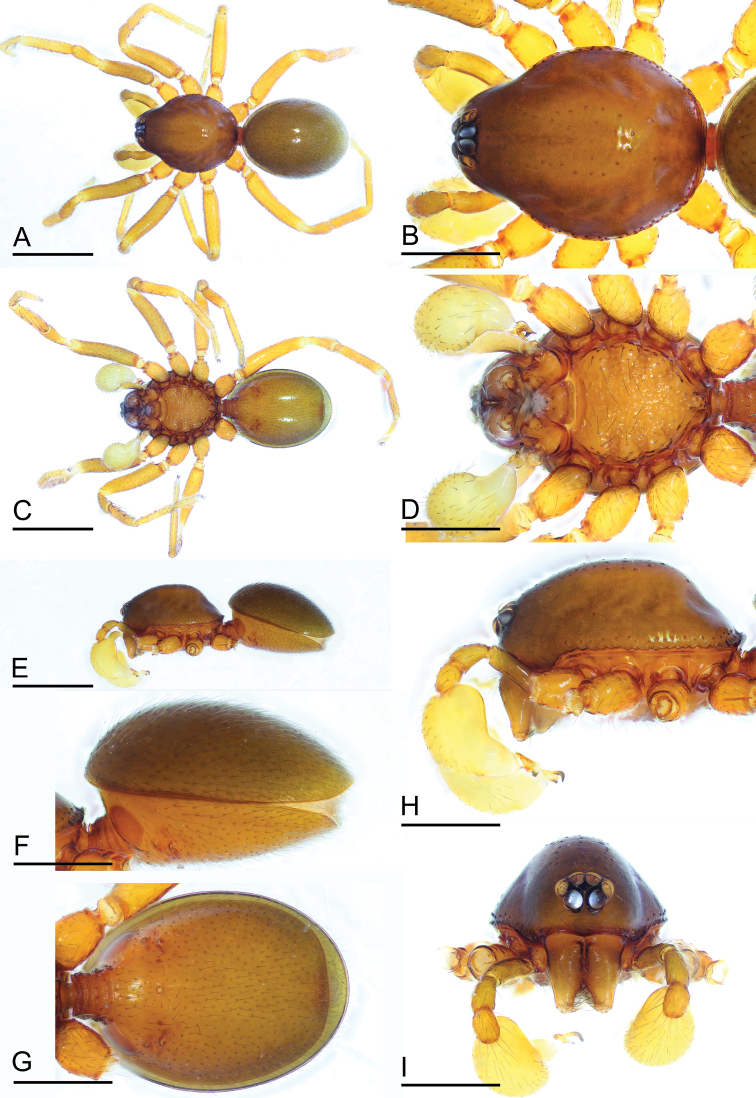
*Paramolotra pome* sp. nov., male holotype (SYNU-435) **A, C, E** habitus in dorsal, ventral, and lateral views **B, D, H, I** prosoma in dorsal, ventral, lateral, and anterior views **F, G** abdomen in lateral and ventral views. Scale bars: 0.8 mm (**A, C, E**); 0.4 mm (**B, D, F–I**).

**Figure 2.**
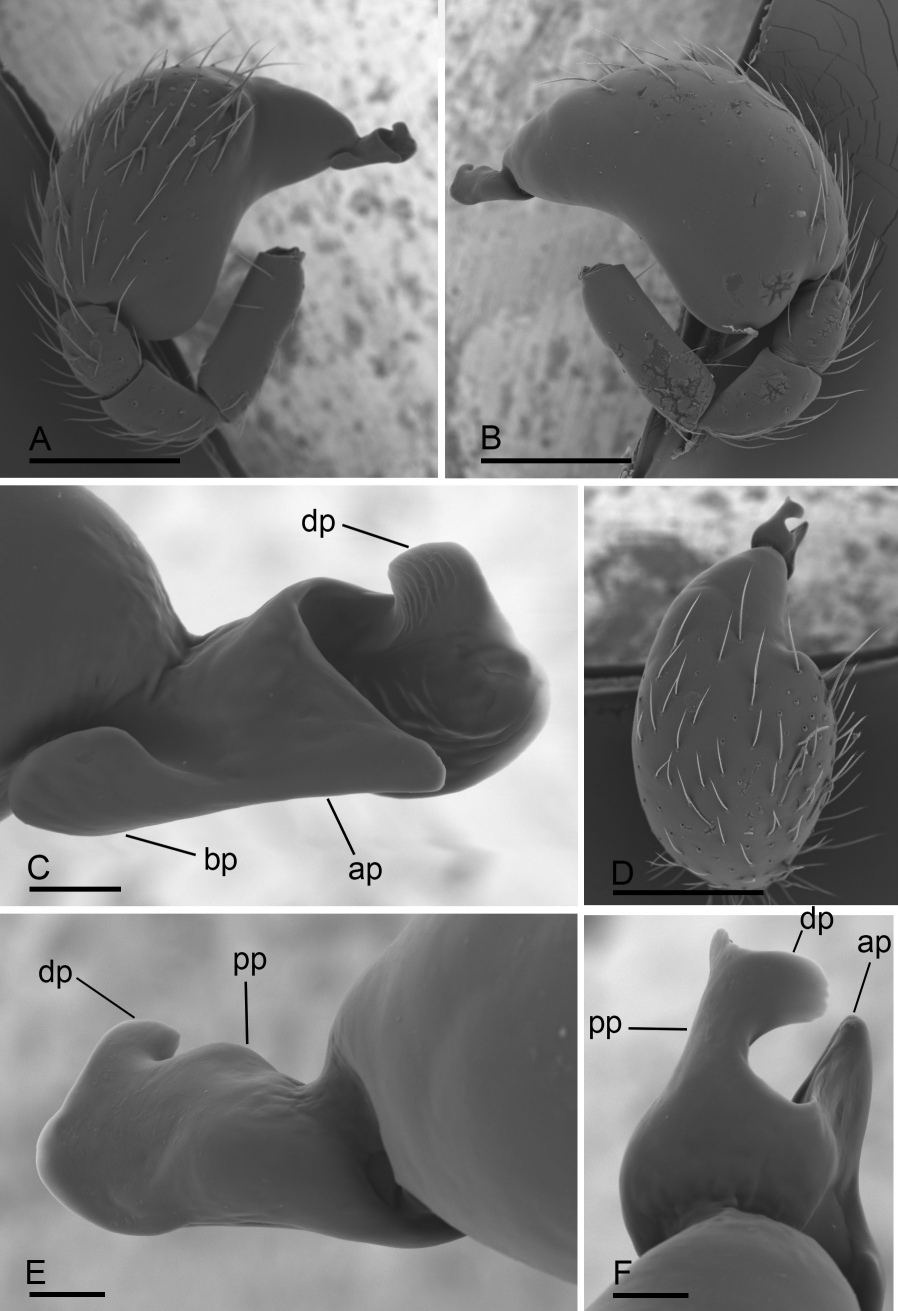
*Paramolotra pome* sp. nov., male left palp, SEM**A, B, D** prolateral, retrolateral, and dorsal views **C, E, F** distal part of cymbiobulbus, prolateral, retrolateral, and dorsal views. Abbreviations: ap = anterior protrusion; bp = basal protrusion; dp = distal protrusion; pp = posterior protrusion. Scale bars: 0.2 mm (**A, B, D**); 0.02 mm (**C, E, F**).

**Figure 3.**
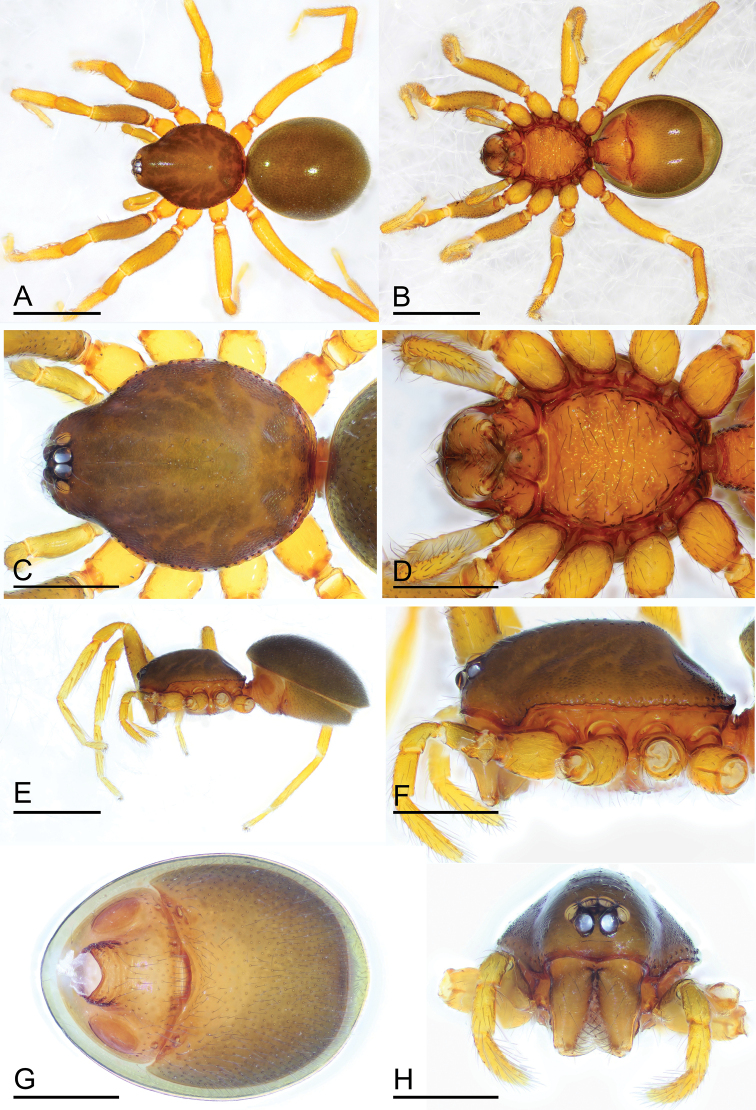
*Paramolotra pome* sp. nov., female paratype (SYNU-438) **A, B, E** habitus in dorsal, ventral, and lateral views **C, D, F, H** prosoma in dorsal, ventral, lateral, and anterior views **G** abdomen in ventral view. Scale bars: 0.8 mm (**A, B, E**); 0.4 mm (**C, D, F–H**).

**Figure 4.**
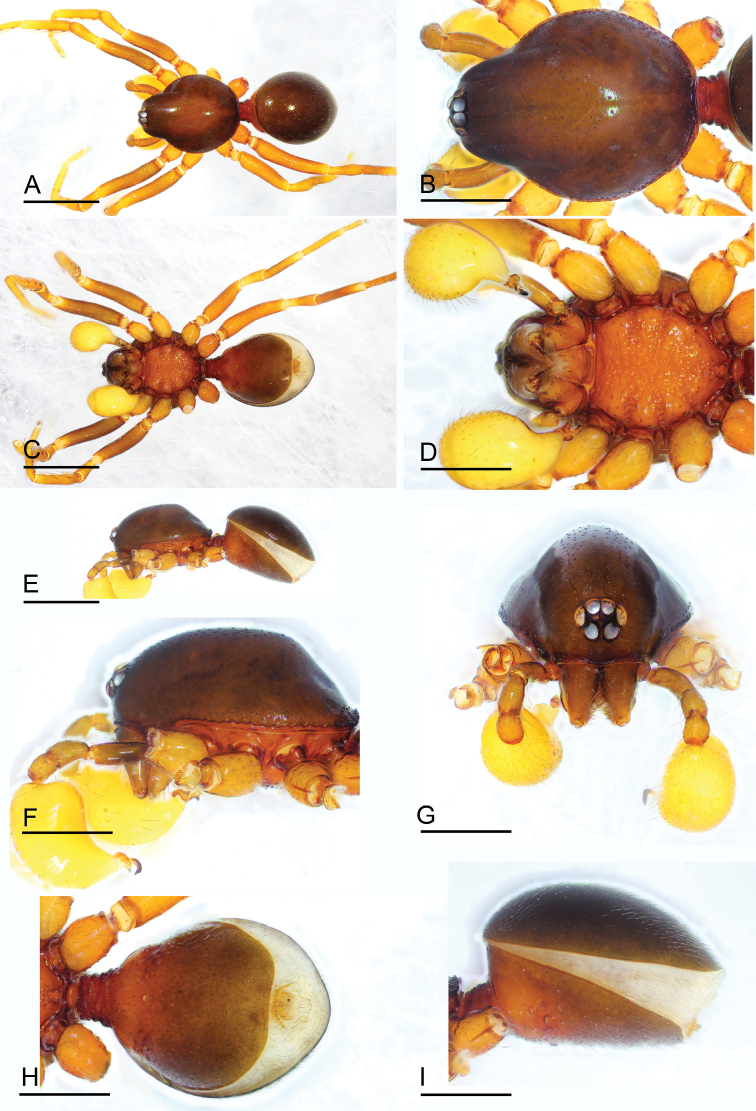
*Paramolotra metok* sp. nov., male holotype (SYNU-441) **A, C, E** habitus in dorsal, ventral, and lateral views **B, D, F, G** prosoma in dorsal, ventral, lateral, and anterior views **H, I** abdomen in ventral and lateral views. Scale bars: 0.8 mm (**A, C, E**); 0.4 mm (**B, D, F–I**).

##### Description.

**Figure 5.**
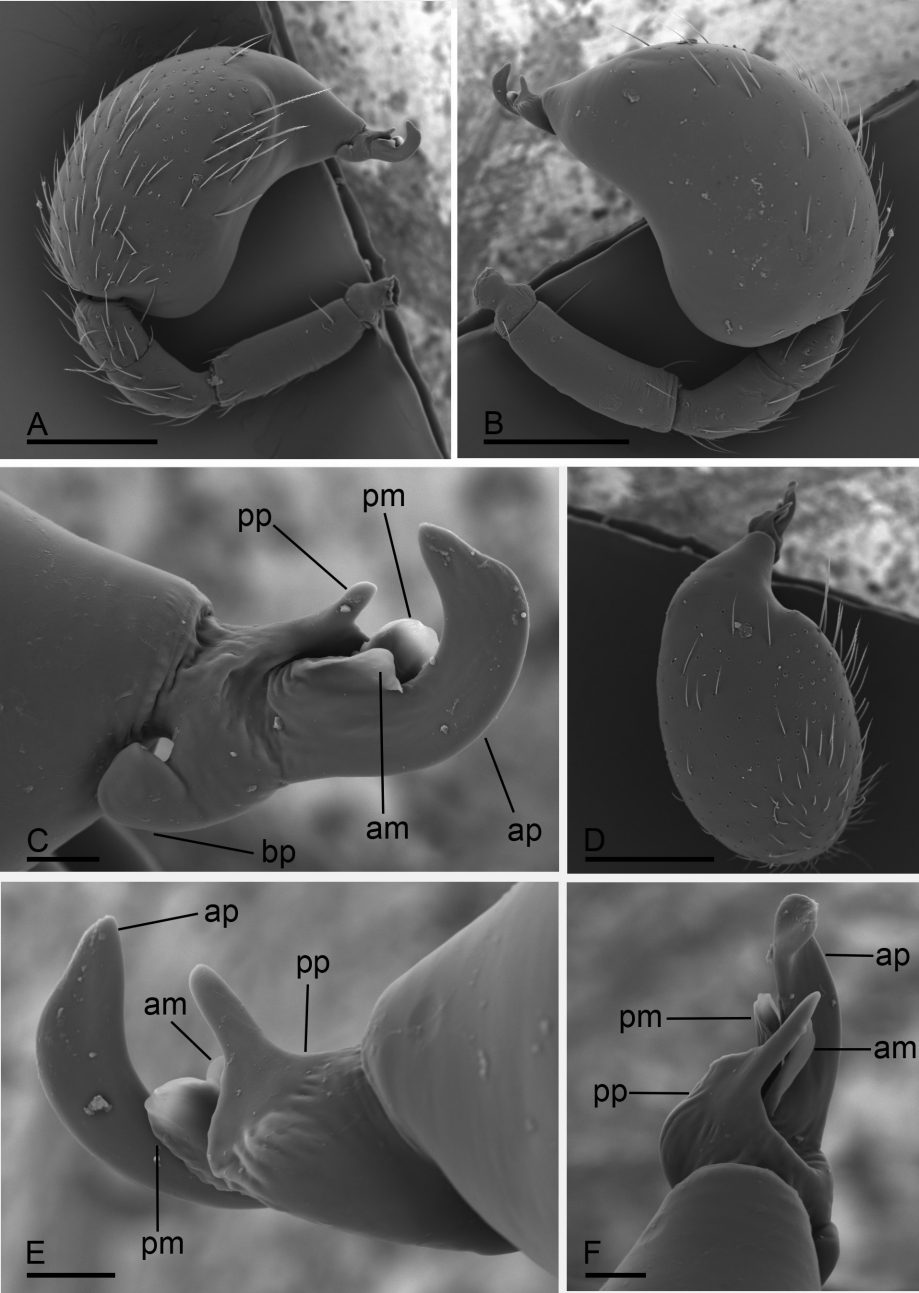
*Paramolotra metok* sp. nov., male left palp, SEM**A, B, D** prolateral, retrolateral, and dorsal views **C, E, F** distal part of cymbiobulbus, prolateral, retrolateral, and dorsal views. Abbreviations: am = anterior membrane; ap = anterior protrusion; bp = basal protrusion; pm = posterior membrane; pp = posterior protrusion. Scale bars: 0.2 mm (**A, B, D**); 0.02 mm (**C, E, F**).

**Male. *Body***: yellow-brown, legs yellow. ***Carapace*** (Figs 1B, H, 4B, F): broadly oval in dorsal view, without any pattern; pars cephalica slightly elevated in lateral view, with rounded posterolateral corners, posterolateral edge without pits, posterior margin not bulging below posterior rim, anterolateral corners without extensions or projections, posterolateral surface without spikes, thorax without depressions, fovea absent, without radiating rows of pits; surface finely reticulated (anterolateral surface striated in *P. metok* sp. nov.), lateral margin with small blunt denticles, marginal setae present. ***Eyes*** (Figs 1B, I, 4B, G): 6, well-developed, arranged in a compact group; ALE largest, PME, PLE subequal; ALE separated by less than their radius, ALE–PLE separated by less than ALE radius, PME touching each other; posterior row straight from above, procurved from front. ***Clypeus*** (Figs 1I, 4G): margin unmodified, sinuous in front view, vertical in lateral view. ***Mouthparts*** (Figs 1I, 4G, 7E–H, 8A–L): chelicerae straight, anterior face strongly swollen, with cone-shaped protuberance in lateral view; with large tooth on promargin; with slit sensillum on distal part of cheliceral paturon; labium rectangular, anterior margin indented, not fused to sternum; endites same as sternum in sclerotization. ***Sternum*** (Figs 1D, 4D): uniformly orange-brown, not fused to carapace; longer than wide, with radial furrows between coxae, surface rugose; setae sparse, dark, needlelike, evenly scattered. ***Abdomen*** (Figs 1F, G, 4H, I): ovoid, rounded posteriorly; booklung covers large, brown, without setae, anterolateral edge unmodified; pedicel tube medium-sized, ribbed, scutum not extending far beyond dorsum of pedicel, lacking plumose hairs; sperm pore small, oval, rebordered, situated between posterior spiracles; anterior and posterior spiracles not connected by grooves; dorsal scutum strongly sclerotized, covering full length of abdomen, no soft tissue visible from above, separate from epigastric scutum; epigastric scutum strongly sclerotized, surrounding pedicel; postgastric scutum strongly sclerotized, covering nearly full length of abdomen, fused to epigastric scutum, with posteriorly directed lateral apodemes; spinneret scutum present as incomplete ring, with fringe of setae; colulus represented only by setae. ***Legs*** (Figs 1A, C, 4A, C): yellowish brown; leg spines: tibiae I–II with 4 pairs of ventral spines; metatarsi I–II with 2 pairs of ventral spines, legs III and IV without spines. ***Palp*** (Figs 2, 5, 7A–D): femur, patella and tibia brown, cymbiobulbus yellow; cymbium completely fused with bulb; embolar region consists of a basal protrusion, an anterior protrusion and a posterior protrusion, which distinctly extend beyond the tip of the cymbiobulbus.

**Figure 6.**
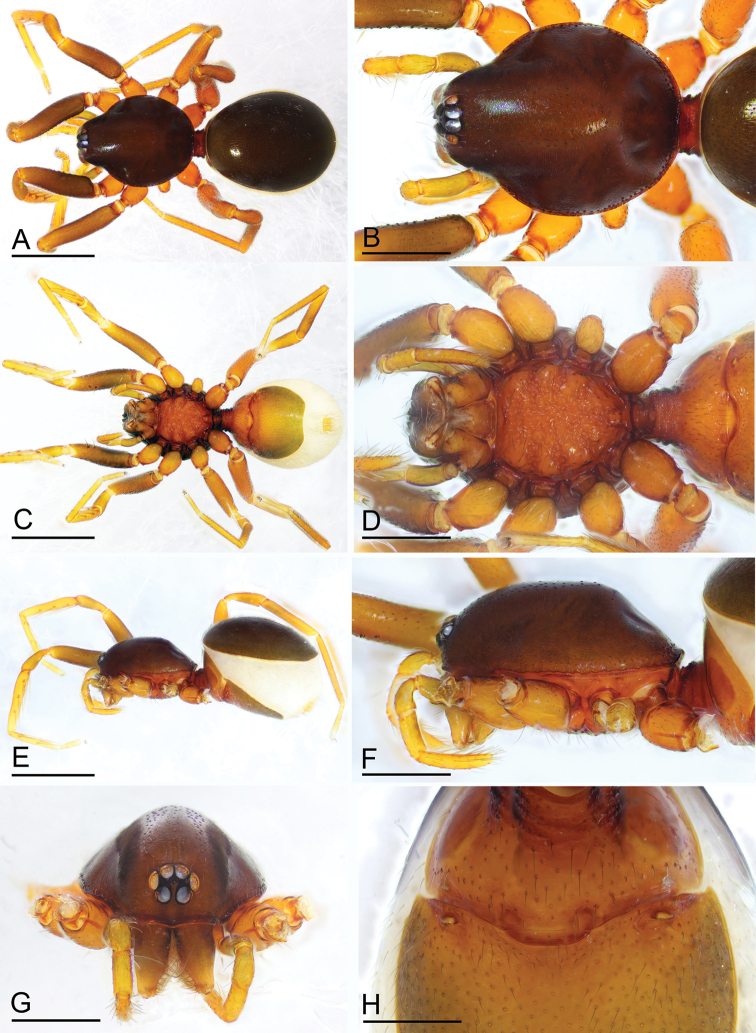
*Paramolotra metok* sp. nov., female paratype (SYNU-443) **A, C, E** habitus in dorsal, ventral, and lateral views **B, D, F, G** prosoma in dorsal, ventral, lateral, and anterior views **H** abdomen in ventral view. Scale bars: 0.8 mm (**A, C, E**); 0.4 mm (**B, D, F, G**); 0.2 mm (**H**).

**Female.** As in male except as noted. Chelicerae without cone-shaped protrusion. Postgastric scutum rectangular, not fused to epigastric scutum. ***Epigastric area*** (Figs 3G, 6H, 9A, C): surface with conspicuous genital atrium. ***Endogyne*** (Fig. 9B, D): with a T-shaped sclerite; apodemes well-developed; receptaculum present.

##### Composition.

*Paramolotra pome* sp. nov. (♂♀), *P. metok* sp. nov. (♂♀).

##### Distribution.

China (Tibet).

#### Paramolotra pome

Taxon classificationAnimaliaAraneaeOonopidae

Tong & Li sp. nov.

58E354EE-A4D7-5072-B7CF-D7E7CAD6BCDD

http://zoobank.org/5F7AF588-99BC-4F35-AE3F-82E62328D0E5

**Figure 7.**
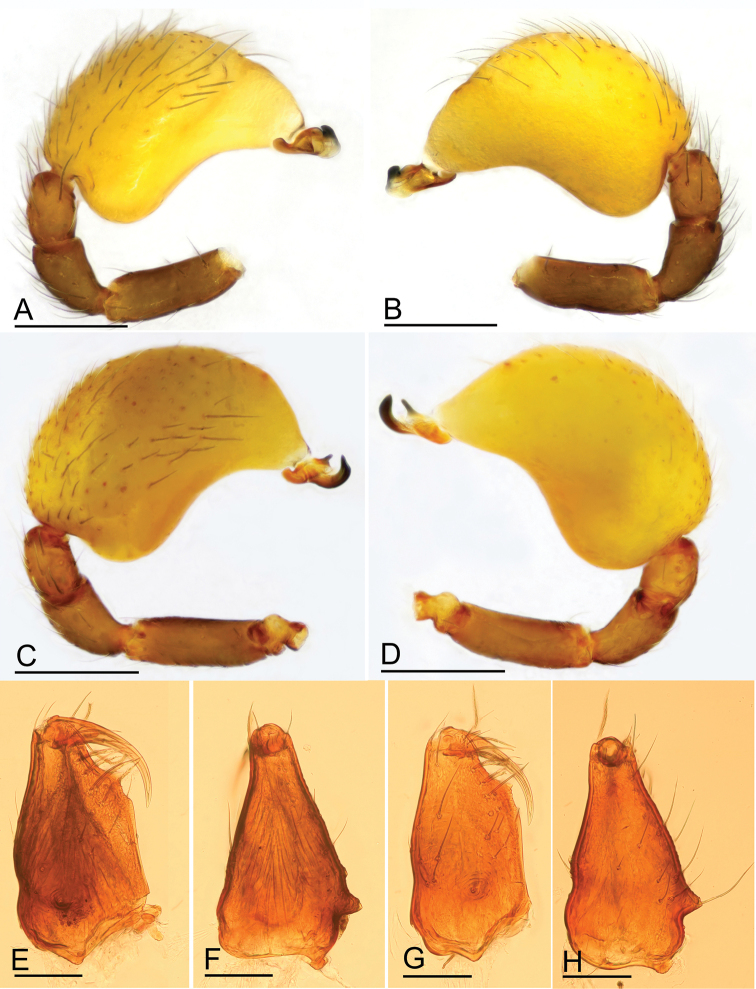
*Paramolotra pome* sp. nov. **A, B, E, F**, male holotype (SYNU-435); *Paramolotra metok* sp. nov. **C, D, G, H** male holotype (SYNU-441) **A, C** left palp, prolateral view **B, D** left palp, retrolateral view **E, G** left chelicerae, anterior view **F, H** left chelicerae, lateral view. Scale bars: 0.2 mm (**A–D**); 0.1 mm (**E–H**).

**Figure 8.**
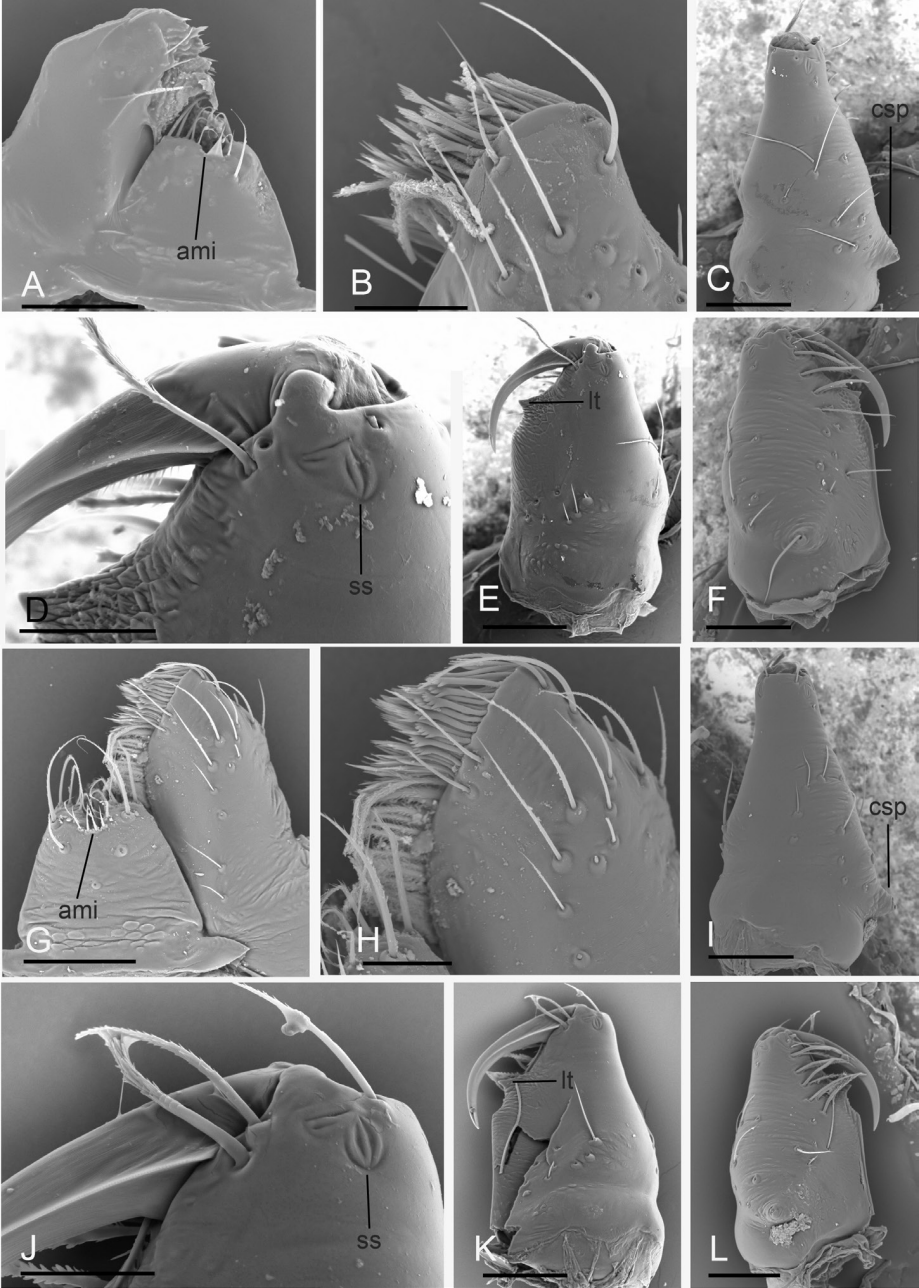
*Paramolotra pome* sp. nov. **A–F** male holotype (SYNU-435); *Paramolotra metok* sp. nov. **G–L** male holotype (SYNU-441) **A, G** labium and endite, ventral view **B, H** left endite, ventral view **C, I, E, K, F, L** left chelicerae, lateral, posterior, and anterior views **D, J** left chelicerae, posterior magnified views. Abbreviations: ami = anterior median indentation; csp = cone-shaped protuberance; lt = large tooth; ss = slit sensillum. Scale bars: 0.1 mm (**A, C, E–G, I, K, L**); 0.04 mm (**B, D, H, J**).

**Figure 9.**
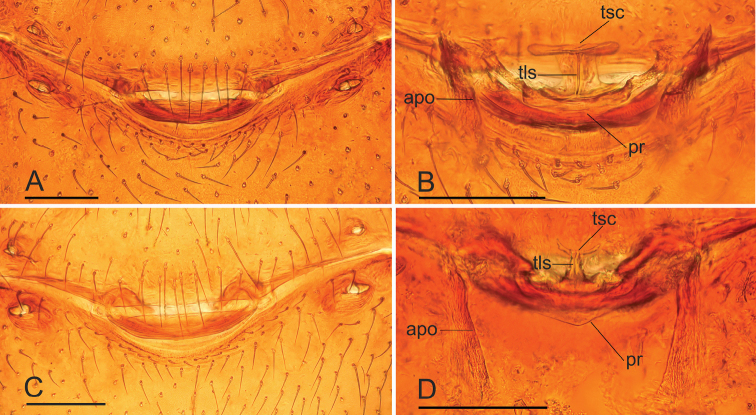
*Paramolotra pome* sp. nov. **A, B** female paratype (SYNU-438); *Paramolotra metok* sp. nov. **C, D** female paratype (SYNU-443) **A, C** copulatory organ, ventral view **B, D** copulatory organ, dorsal view. Abbreviations: apo = apodemes; pr = posterior receptaculum; tls = tube-like structure; tsc = T-shaped sclerite. Scale bars: 0.1 mm.

**Figure 10.**
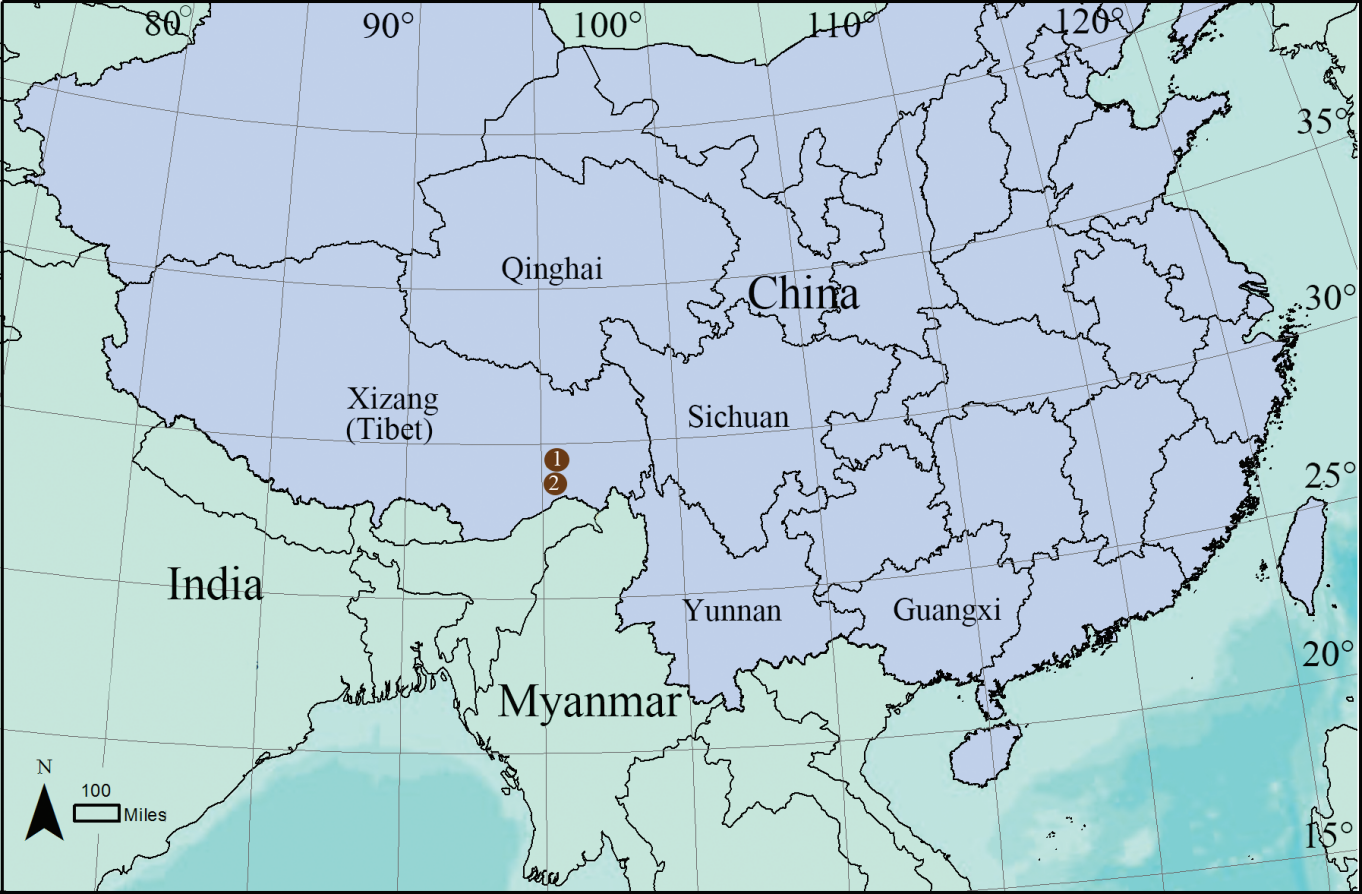
Distribution records of *Paramolotra* species from Tibet, China **1**. *P. pome* sp. nov. **2**. *P. metok* sp. nov.

Figures 1 , 2 , 3 , 7A, B, E, F , 8A–F , 9A, B , 10

##### Type materials.

***Holotype*** ♂: China, Tibet, Nyingchi, Pome County, road to Metok County, 80 K; 29°39.897'N, 95°29.963'E; 2140 m a.s.l.; 10.VIII.2013; Y. Lin leg. (SYNU-435). ***Paratypes*** 2♂3♀: same data as for holotype (SYNU-436-440).

##### Diagnosis.

This new species is similar to *Paramolotra metok* sp. nov., but can be distinguished by the narrow anterior protrusion (ap) and broad posterior protrusion (pp) of embolar region (Fig. 2), and the long, straight arms of T-shaped sclerite of endogyne (Fig. 9B). *Paramolotra metok* sp. nov. males have hook like anterior protrusion (ap) and basally broad, distally finger like posterior protrusion (pp) of embolar region (Fig. 5), and females have very short anterior arm of T-shaped sclerite of endogyne (Fig. 9D).

##### Description.

**Male (holotype). *Body***: carapace brown, abdomen light brown; habitus as in Fig. 1A, C, E; body length 2.15. ***Carapace*** (Fig. 1B, H): 1.04 long, 0.80 wide. ***Clypeus*** (Fig. 1I): ALE separated from edge of carapace by 1.1 times their diameter. ***Abdomen*** (Fig. 1F, G): 1.06 long, 0.82 wide. ***Palp*** (Figs 2, 7A, B): femur 0.25 long, patella 0.18 long, tibia 0.12 long, cymbiobulbus 0.57 long, 0.33 wide, length/maximal width = 1.58; embolar region with a broad basal protrusion (bp), a narrow anterior protrusion (ap), a broad posterior protrusion (pp) and a distal dorsal protrusion (dp).

**Female (paratype, SYNU-438).** As in male except as noted. ***Body***: habitus as in Fig. 3A, B, E; body length 2.24. ***Carapace*** (Fig. 3C, F, H): 1.06 long, 0.82 wide. ***Abdomen*** (Fig. 3G): 1.24 long, 0.97 wide. ***Epigastric area*** (Figs 3G, 9A): genital atrium relatively wide, broadly oval. ***Endogyne*** (Fig. 9B): with a large T-shaped sclerite; the anterior arm of the T-shaped sclerite long and strong.

##### Etymology.

The specific name is a noun in apposition taken from the type locality.

##### Distribution.

Known only from the type locality (Fig. 10).

#### Paramolotra metok

Taxon classificationAnimaliaAraneaeOonopidae

Tong & Li sp. nov.

4EDF8C4F-A0B9-5EFD-A719-905B72978BDF

http://zoobank.org/39AD2A01-EBD1-4798-A126-39ECB8E0D596

Figures 4 , 5 , 6 , 7C, D, G, H , 8G–L , 9C, D , 10

##### Type materials.

***Holotype*** ♂: China, Tibet, Nyingchi, Metok County, Metok Town; 29°19.399'N, 95°20.448'E; 1300 m a.s.l.; 3.VIII.2013; Y. Lin leg. (SYNU-441). ***Paratypes*** 1♂2♀: same data as for holotype (SYNU-442-444); 1♀: same data as for holotype (SYNU-446); 1♀: Metok County, Yadang Village; 29°20.605'N, 95°20.807'E; 1360 m a.s.l.; Y. Lin leg. (SYNU-445); 2♀: Metok County, Metok Town, Countryside Tour, back hills; 29°19.087'N, 95°18.876'E; 1280 m a.s.l.; Y. Lin leg. (SYNU-447-448); 1♂: Metok County, Metok Town; 29°19.382'N, 95°19.016'E; 980 m a.s.l.; Y. Lin leg. (SYNU-449).

##### Diagnosis.

This new species is similar to *Paramolotra pome* sp. nov., but can be distinguished by the long, curved hook like anterior protrusion (ap) of embolar region (Fig. 5), and the very short anterior arm of T-shaped sclerite of endogyne (Fig. 9D). *Paramolotra pome* sp. nov. males have the narrow anterior protrusion (ap) of embolar region (Fig. 2), and females have very long anterior arm of T-shaped sclerite of endogyne (Fig. 9B).

##### Description.

**Male (holotype). *Body***: dark brown; habitus as in Fig. 4A, C, E; body length 2.16. ***Carapace*** (Fig. 4B, F): 1.06 long, 0.87 wide. ***Clypeus*** (Fig. 4G): ALE separated from edge of carapace by 1.3 times their diameter. ***Abdomen*** (Fig. 4H, I): 0.99 long, 0.79 wide. ***Palp*** (Figs 5, 7C, D): femur 0.24 long, patella 0.17 long, tibia 0.13 long; cymbiobulbus 0.52 long, 0.28 wide, length/maximal width = 1.86; embolar region with an ear-shaped basal protrusion (bp), a long, curved hook like anterior protrusion (ap), a basally broad, distally finger like posterior protrusion (pp), an anterior membrane (am) and a posterior membrane (pm).

**Female (paratype, SYNU-443).** As in male except as noted. ***Body***: length 2.48; habitus as in Fig. 6A, C, E. ***Carapace*** (Fig. 6B, F, G): 1.12 long, 0.89 wide. ***Abdomen***: 1.28 long, 0.98 wide. ***Epigastric area*** (Figs 6H, 9C): genital atrium relatively wide, broadly oval. ***Endogyne*** (Fig. 9D): with a T-shaped sclerite; the anterior arm of the T-shaped sclerite very small.

##### Etymology.

The specific name is a noun in apposition taken from the type locality.

##### Distribution.

Known only from the type locality (Fig. 10).

## Discussion

The homologies in the genitalia of males and females of *Paramolotra* gen. nov. are unclear. *Paramolotra* gen. nov. are very similar to *Promolotra* in the fused palpal bulb and cymbium and the somatic characters. The embolar region of *Promolotra* has 3 leaf-like, wrinkled texture, and nearly translucent lobes (Tong and Li 2020: figs 1H–J, 5H–J), which is different from the sclerotized protrusions of the embolar region (Fig. 7A–D) of *Paramolotra* gen. nov. The endogyne of *Promolotra* has a horseshoe-shaped sclerite (Tong and Li 2020: fig. 4G), which is lacking in the new genus. However, the T-shaped sclerite and the tube-like structure of endogyne (Tong and Li 2020: figs 4G; 9B, D) are quite homogeneous.

As for the other Asian oonopid genera, i.e., *Kachinia* Tong & Li, 2018, and *Vientianea* Tong & Li, 2013, also have heavily sclerotized abdominal scuta and leg spines. *Paramolotra* gen. nov. are quite different from both genera. The genus *Kachinia* differs from *Paramolotra* gen. nov. by the heavily sclerotized and darkened palps of males (Tong et al. 2018: figs 1I–K, 4I–K), and the tube-like posterior receptacle of endogyne (Tong et al. 2018: figs 3I, J, 6I, J). The genus *Vientianea* differs from *Paramolotra* gen. nov. by the enlarged male palpal patella (Tong and Li 2013: figs 24, 25), and the medially stick-shaped sclerite and the strongly curved circular sclerite of endogyne (Tong and Li 2013: figs 23, 33). So, *Paramolotra* gen. nov. and *Promolotra* maybe represent a different genus group in Asia.

## Supplementary Material

XML Treatment for Paramolotra

XML Treatment for Paramolotra pome

XML Treatment for Paramolotra metok
